# Antioxidative, anti-inflammatory and anti-apoptotic effects of ellagic acid in liver and brain of rats treated by D-galactose

**DOI:** 10.1038/s41598-018-19732-0

**Published:** 2018-01-23

**Authors:** Peng Chen, Fuchao Chen, Benhong Zhou

**Affiliations:** 1 Department of Pharmacy, Renmin Hospital of Wuhan University, Wuhan 430060, Hubei Province, P.R, China; 2 School of Pharmaceutical Sciences, Wuhan University, Hubei Wuhan, 430071, P.R China; 3 Department of Pharmacy, Dongfeng Hospital, Hubei University of Medicine, Shi yan, Hubei 442008, P.R, China

**Keywords:** Diagnostics, Risk factors

## Abstract

Accumulating evidence has suggested that oxidative stress and apoptosis are involved in the ageing process. D-galactose (gal) has been reported to cause symptoms of ageing in rats, accompanied by liver and brain injuries. Our study aimed to investigate the potential antioxidative, anti-inflammatory and anti-apoptotic effects of ellagic acid and to explore how these effects act on rats in a D-gal-induced ageing model. Ageing was induced by subcutaneous injection of D-gal (100 mg/kg/d for 8 weeks). Ellagic acid was simultaneously administered to the D-gal-induced ageing rats once daily by intragastric gavage. Finally, the mental condition, body weight, organ index, levels of inflammatory cytokines, antioxidative enzymes, and liver function, as well as the expression of pro- and anti-apoptotic proteins, were monitored. Our results showed that ellagic acid could improve the mental condition, body weight, organ index and significantly decrease the levels of inflammatory cytokines, normalize the activities of antioxidative enzymes, and modulate the expression of apoptotic protein in ageing rats. In conclusion, the results of this study illustrate that ellagic acid was suitable for the treatment of some ageing-associated problems, such as oxidative stress, and had beneficial effects for age-associated diseases.

## Introduction

Ageing is a natural phenomenon and is associated with a variety of chronic diseases such as cancer, retinopathy, atherosclerosis and Parkinson’s disease^1^. Anti-ageing has become an important issue with the increase in the elderly population worldwide.

The free radical theory poses that reactive oxygen species (ROS)-induced oxidative damage plays a requisite part in the pathophysiology of ageing^2,3^. Abundant evidence suggests that oxygen-derived free radicals are closely correlated to signal recognition, protein expression, and immune response. However, excess ROS have detrimental effects on humans, including DNA damage, increasing membrane lipid peroxidation, and activating apoptosis, eventually leading to cellular injury^4,5^. In addition, it has been reported that supplementation with antioxidants can lead to the scavenging of free radicals, which can slow the ageing process^6^.

Recently, plenty of experimental and clinical data have supported the notion that the chronic administration of D-galactose can accelerate ageing. D-gal is a reducing sugar also known as a physiological nutrient, which can be combined with the free amines of amino acids in proteins through nonenzymatic glycation to form advanced glycation end products^7,8^. As such, excessive complementation of D-gal is likely to lead to the accumulation of ROS through the oxidative metabolism of D-gal, as well as through glycation end products. Interestingly, chronic administration of D-gal for a period of 6–10 weeks in rodents was reported to show an increased generation of free radicals in the liver and brain^9,10^. Furthermore, it was demonstrated that oxidative damage was also associated with inflammatory damage and apoptosis in D-gal-exposed rats. Therefore, D-gal-treated rats have been gradually used as animal models for anti-ageing or organ injury studies^11^.

Ellagic acid (C_14_H_6_O_8_,2,3,7,8-tetrahydroxybenzopyrano-[5, 4, 3-cde] benzopyran-5, 10-dione, EA), a type of polyphenolic compound, is widely distributed in crude drugs such as Galla Chinensis and Pericarpium Granati and has been demonstrated to posses a strong ability to scavenge free radicals both *in vivo* and *in vitro*^12–14^. In addition, EA has long been considered to have anti-apoptosis and anti-inflammatory activities in many magnocellular systems. As mentioned previously, many studies have suggested that EA has strong antioxidant activities and anti-ageing effects^15–17^. However, to our knowledge, there are no reports to date about the protective effects of EA on D-gal-induced ageing of rat liver and brain, nor have there been reports regarding its underlying anti-ageing molecular mechanisms^18^. Thus, the present study aims to use D-gal-injected rats to evaluate the possible hepatoprotective and cerebroprotective effects of EA and explore the underlying mechanisms *in vivo*.

## Materials and Methods

### Chemicals and reagents

**Figure 1:**
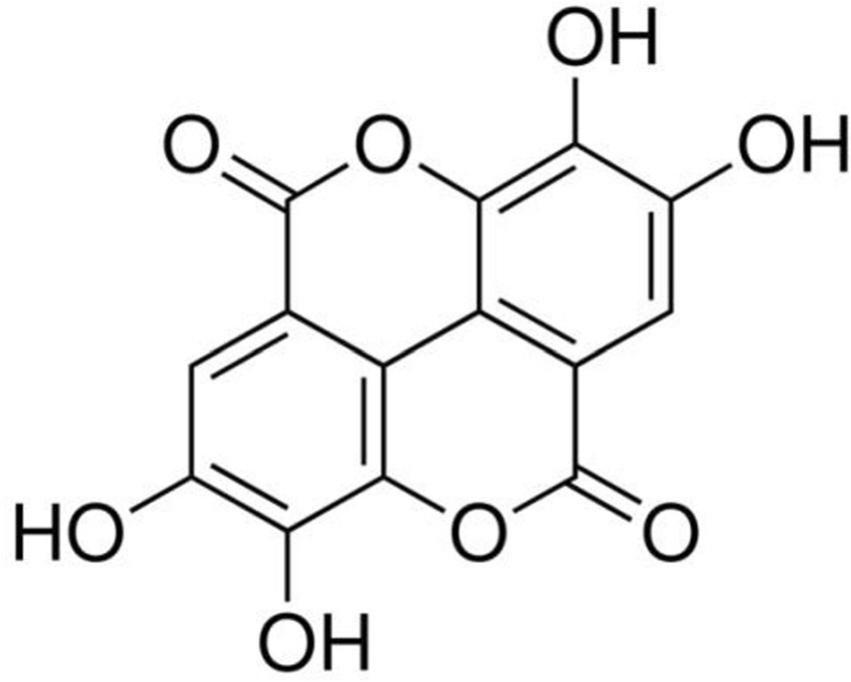
Chemical structure of EA.

Ellagic acid standard (Fig. 1, Lot No. C1502030, purity > 98%) was supplied by Aladdin Reagents (Shanghai, China). D-gal was purchased from Sigma Chemical (St. Louis, MO, USA, No. 100151-230, purity ≧ 99%). Vitamin E (VE) was supplied by Guoyao Chemical Reagent Co., LTD. (Shanghai, China, No. 20161222, purity > 98%).

Assay kits for the measurements of aspartate aminotransferase (AST, No. 20170632), alanine aminotransferase (ALT, No. 20170531), malondialdehyde (MDA, No. 201705 24), superoxide dismutase (SOD, No. 20170321), glutathione peroxidase (GSH-Px, No. 20170516), catalase (CAT, No. 20170520) and total antioxidant capacity (T-AOC, No. 20170412) were obtained from Nanjing Jiancheng Biological Engineering Research Center (Nanjing, China). ELISA assay kits for tumour necrosis factor-α (TNF-α, No. 20170512), Interleukin-1 beta (IL-1 β, No. 20170512) and Interleukin-6 (IL-6, No. 20170512) were purchased from Shanghai Jin Ma Laboratory Equipment, Co., LTD. (Shanghai, China). The antibodies against Bax, Bcl-2 and caspase-3 were purchased from Santa Cruz Biotechnology, Inc. (Dallas, TX, USA). All other solvents and reagents used in the study were at least of analytical grade.

### Preparation of the D-gal-treated rats and drug administration

The male Sprague-Dawley (SD) rats, aged 4 weeks (180–200 g), were obtained from the Center of Experimental Animals of Medical College, Wuhan University. The committee for experimental animals of Wuhan University approved all experimental procedures, and the procedures complied with the Guidelines for the Care and Use of Laboratory Animals. The approved protocol number is SCXK (X) 2013–0004. The experimental animals were housed at 20 °C with 12-h light-dark cycles. After one week of acclimatization, animals were randomly divided into six groups (8 rats per group, n = 8) including the blank control group, the model group (D-gal), the positive control group (VE, 150 mg/kg), the high-dose EA-treated group (EA-H, 150 mg/kg), the medium-dose EA group (EA-M, 100 mg/kg) and the low-dose EA-treated group (EA-L, 50 mg/kg). Except for the blank control group, all groups received subcutaneous injectios of D-gal (100 mg/kg/d for 8 weeks), which was dissolved in normal saline solution (0.9%, w/v)^19–22^. The rats in the EA and VE groups (Tween 80 was used to dissolve VE and EA reagents) were subcutaneously and orally gavaged, while the rats in the control group were treated with hypodermic injections of 0.9% normal saline (100 mg/kg) at equal volumes and concomitantly administered normal saline by gavage^23^. The animals were monitored daily for their food and water intake before and after injection. The selection of the dose of EA in our study was based on our preliminary experimenta tion and previous investigation^24^. All animal procedures were conducted in accordance with the China Animal Welfare Legislation and were approved by the Ethics Committee on the Care and Use of Laboratory Animals in Wuhan University (Wuhan, China).

### Assay of body weight and organ indices

At the end of the experiment, we collected inferior vena cava blood, heart, liver, brain and kidney under ether anaesthesia. Parts of the fresh brain and liver tissues were removed and kept frozen for biochemical measurements and histopathological assays. After the rats were sacrificed, we isolated the spleens, thymuses, and kidneys from the bodies and then weighed these organs to calculate the organ coefficients. The organ coefficient of an animal is defined as the ratio of organ weight in the medium to to tal body weight, according to the following equation: coefficient (mg/g) = organ weight (mg)/body weight(g)^25^.

### Histological Studies

Each sample from the different organs was divided into two parts: (1) fixed in 10% formalin solution for 12 h, embedded in paraffin and stained with haematoxylin and eosin (H&E), and (2) cut into blocks l × l × l mm^3^ in size, fixed in 4% glutaraldehyde, cleaned repeatedly with PBS, fixed for 2 h continuously after treating with osmic acid, dyed with saturated uranium acetate for one night, dehydrated with 50%, 70%, 90%, and 100% ethanol, soaked with a solution of epoxy propane:resin (1:1), and embedded, aggregated and examined microscopically^26^.

### Determination of Antioxidant activity

For biochemical analysis, animals were heavily anaesthetized and then sacrificed. The organs of rats were promptly dissected and perfused with 50 mM (pH 7.4) of icecold phosphate-buffered saline solution (PBS). Then, the organs were washed with physiological saline (w/v = 1:4) to remove the blood, and homogenates of 10% were prepared in 5% (w/v) potassium chloride using a homogenizer^27^. The homogenates were centrifuged at 8000 × g for 10 minutes in a high-speed refrigerated centrifuge at 4 °C, and the supernatants were used for further biochemical analysis. The protein concentrations were measured by the BCA (bicinchoninic acid) method using bovine serum albumin as a standard. The extent of systemic oxidative stress in serum, brains, and livers of rats was measured by detecting the activities of SOD, CAT, T-AOC, and GSH-Px, as well as the levels of MDA, according to the manufacturer’s protocol.

### Evaluation of AST and ALT in liver

The levels of AST and ALT in the livers were assessed by an ELISA assay kit (Nanjing, China) in accordance with the manufacturer’s instructions. The prevention rate (%) was defined as follows: (the level of AST or ALT (model group) - the level of AST or ALT (dosing group))*100/the level of AST or ALT (model group).

### Analysis of inflammatory cytokines in the serum

Blood samples were collected from the vena cavas of rats under ether anaesthesia into heparin-coated microcapillaries. The samples were placed in centrifuge tubes and centrifuged at 5000 rpm for 20 min at 4 °C. The presence of TNF-α, IL-1β and IL-6 in the cell supernatant was measured with a rat standard-ELISA kit. The results were calculated based on the absorbance levels of complex cytokine-antibodies, and the units of cytokines were described as pg/ml.

### Western blotting analysis

The animal tissue homogenates or cells were lysed by a RIPA (radioimmunopre cipitation assay) lysis buffer, which enables the extraction of cytoplasmic, membrane and nuclear proteins. The concentration of protein was determined by the BCA protein assay kit. Tissue protein samples were separated by 10% SDS-polyacrylamide gel electrophoresis and then transferred onto a PVDF membrane by electrophoretic transfer. The membranes were first incubated in blocking solution (5% (w/v) non-fat milk in tris-buffered saline containing 0.1% (v/v) Tween 20, Triton X-100 (TBST)) and then incubated overnight at 4 °C with different primary antibodies: anti-Bcl-2 (1:1000), antiBax (1:1000), and anti-case-3(1:1000). After washing 3 times with TBST, the membranes were incubated with the peroxidase-conjugated secondary antibody at room temperature for 1 h. Signals were visualized using a GE ImageQuant LAS4000mini, quantified using densitometry with the Gel Doc XR System (Bio-Rad Laboratories, Hercules, CA, USA), and analysed using Quantity One Protein Analysis Software (BioRad Laboratories, Hercules, CA, USA). β-actin was used as an internal control to normalize the relative protein levels.

### Statistical analysis

The results of study were expressed as the mean ± standard deviation (SD). The statistical analyses were performed using the SPSS version 15.0 statistical software package (SPSS Inc., Chicago, IL, USA). The comparisons between different groups were performed using two-way analysis of variance (ANOVA) and least significant difference tests to make a judgement on whether the results were significant. *P* values of less than 0.05 or 0.01 were considered significant.

## Result

### Effect of EA on mental outlook, body weight and organ indices

The rats in the blank control group appeared to shed hair but with no depression or drowsiness from the 6th week. Compared with the blank group, the D-gal group exhibited hair loss from the 2nd week of administration of D-gal, and other significant symptoms of ageing, such as dullness and decreased activity, were aggravated in the 4th week. However, these symptoms decreased in rats of the positive control group and the three EA-dosed groups. The observations showed that when the experiment ended, compared with that of the blank control group, there was a significant decrease in the body weights of rats that were administered D-gal (*P* < 0.05). As shown in Table 1, after 8 weeks of D-gal injection, the organ indices of the spleens, thymuses and kidneys of the D-gal-induced ageing model group increased compared with rats in the blank control group (all *P* < 0.05). In contrast, the administration of VE or EA could attenuate these increases when compared to those in the D-gal-induced ageing model group (*P* < 0.05 or *P* < 0.01).

**Table 1:** Effects of EA on body weight and organ index in D-gal-treated rats

Group	Body weight(g)	Organ index (mg/g)		
		Spleen index	Thymus index	Kidney index
Blank control	36.82 ± 1.55	1.87 ± 0.23	1.45 ± 0.21	10.68 ± 0.56
D-gal	29.06 ± 2.24^△^	1.54 ± 0.24^△^	1.16 ± 0.15^△^	8.01 ± 1.32^△^
VE (150 mg/kg)	34.96 ± 2.67**	1.78 ± 0.15**	1.39 ± 0.18**	9.95 ± 1.29*
EA-L (50 mg/kg)	32.4 ± 2.80*^△^	1.55 ± 0.28*^△^	1.18 ± 0.14^△^	8.29 ± 0.89^△^
EA-M (100 mg/kg)	35.03 ± 2.26**	1.86 ± 0.19**	1.45 ± 0.19**	9.10 ± 0.94*
EA-H (150 mg/kg)	36.74 ± 1.49**	1.98 ± 0.33**	1.47 ± 0.15**	9.86 ± 0.80*

Notes: Dates are given as mean ± SD (n = 8). ^△^P < 0.05 and ^△△^P < 0.01 vs blank control

group. *P < 0.05 and **P < 0.01 vs D-gal model group.

EA-H: high-dose EA-treated group, EA-M: the medium-dose EA group.

EA-L: the low-dose EA-treated group.

### Effect of EA on biochemistry indices in the liver and brain

**Figure 2:**
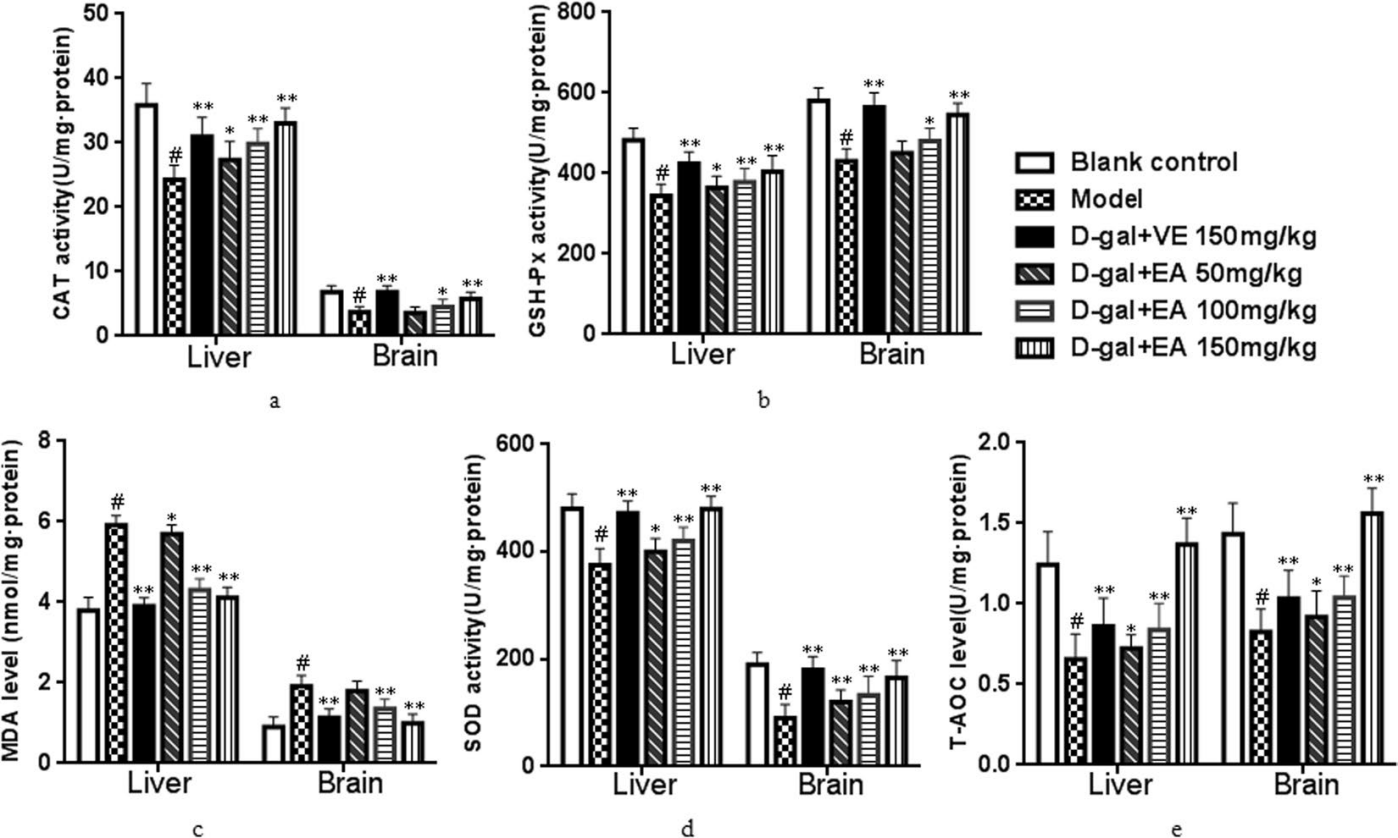
Effects of EA on the activities of CAT (**a**), GSH-Px (**b**), MDA (**c**), SOD (**d**) and T-AOC(e) in rats. Date are presented as mean ± SD from each group(n = 8). ^#^P < 0.01 vs blank control group. **P < 0.01 and *P < 0.05 vs D-gal model group.

Studies have shown that antioxidant levels play a crucial role in the biological ageing process. As shown in Fig. 2(a)–(e), the CAT, GSH-Px, MDA, SOD and T-AOC activities of the liver and brain tissues in the D-gal group showed significant decreases (all *P* < 0.05) compared to the control group. However, the reductions in the activities of these biochemical indices in the liver and brain tissues could be significantly inhibited by treating the ageing rats with EA (EA + D-gal-induced ageing model group). In addition, similar effects were observed in the VE-treated group. The levels of CAT, GSH-Px, SOD and T-AOC in the brain in the 50 mg/kg EA-group rats also increased to some extent, but there was no significance compared with the D-Gal model group (*P* > 0.05). In addition, CAT levels in the liver showed a remarkable increase at a dose of 50 mg/kg of EA, but the difference was not significant (*P* > 0.05). In this study, the results showed that when restoring the antioxidant defence system using a low dose of EA, there was a clearer effect in the liver than in the brain^28^.

The MDA contents in the liver and brain tissues of model rats showed a significantly higher percentage (56.8% and 59.4%, respectively) (both *P* < 0.05) compared with the control group. In contrast, pretreatment with EA (100 or 150 mg/kg) alleviated this effect, resulting in significant decreases in MDA levels in both the liver and the brain (Fig. 2c, both *P* < 0.01).

### Evaluation of pro-inflammatory cytokines

**Figure 3:**
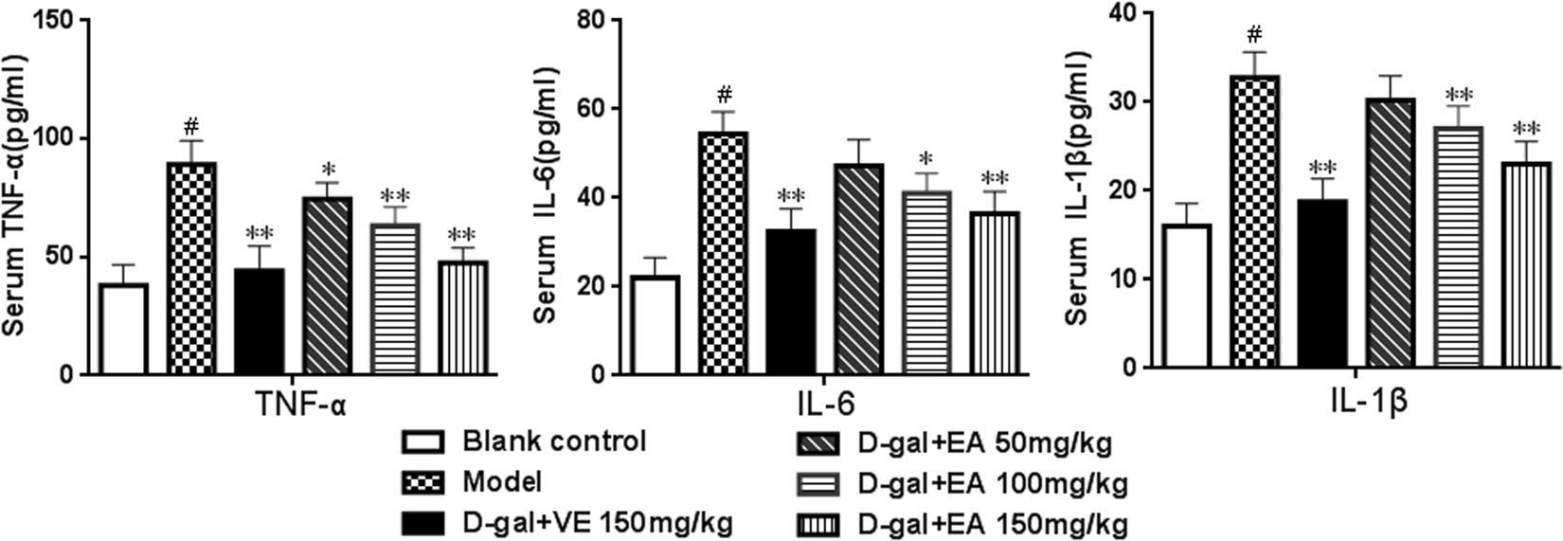
Effects of EA on the levels of TNF-α, IL-6 and IL-1β in sera of rats. The levels of pro-inflammatory were determined by ELISA kits. Values are given as mean ± SD from each group(n = 8). ^#^P < 0.01 vs blank control group. **P < 0.01 and *P < 0.05 vs D-gal model group.

Compared with the control group, the amounts of three pro-inflammatory cytoki-nes including TNF-α, IL-6 and IL-1β in the serum of D-gal-induced ageing rats were markedly increased (by 212.6%, 238.5% and 207.8%, respectively) (Fig. 3, all *P* < 0.0 5), which suggests that the administration of D-gal caused a significant systemic and local chronic inflammatory condition in rats. Interestingly, our results showed that treatment with different concentrations of EA (100 or 150 mg/kg) for eight weeks could significantly weaken the expression of these three pro-inflammatory cytokines (both *P* < 0.05). Additionally, the group administered with 50 mg/kg of EA experienced alleviation in the increased levels IL-6 and IL-1β; however, no significant differences were found when compared with the blank control group (*P* > 0.05). Our findings revealed that EA markedly alleviates the inflammatory state induced by D-gal.

### Measurement of ALT and AST levels

**Figure 4:**
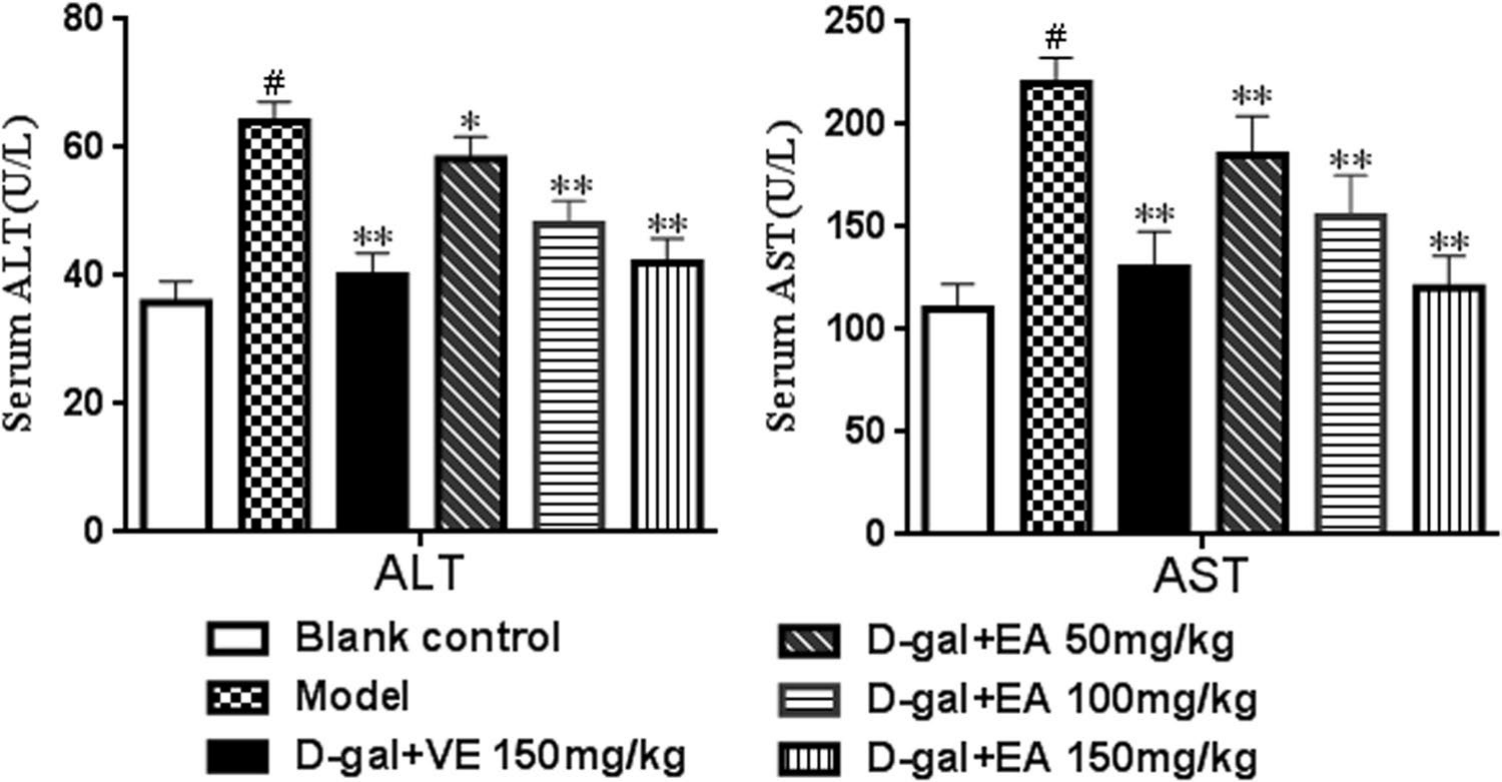
Effect of EA treatment on on the levels of ALT and AST in sera of rats. Date are presented as mean ± SD from each group(n = 8). ^#^P < 0.01 vs blank control group. **P < 0.01 and *P < 0.05 vs D-gal model group.

ALT and AST levels in sera were measured to assess damage to the liver following D-gal treatment. As depicted in Fig. 4, compared with the blank control group, the D-gal-treated group showed a clear increase in the levels of ALT and AST (both *P* < 0.01), which suggests that D-gal induces liver function decline. Rats treated with EA (150 mg/kg) alone experienced a significant decrease in the levels of ALT and AST compared with the D-gal model group (31.5% and 48.6% reductions, respectively). In addition, the increases in the levels of ALT and AST were significantly inhibited by all three tested doses of EA (*P* < 0.05 or *P* < 0.01). The results showed that EA could alleviate the liver damage induced by D-gal on rats.

### Analysis of histopathological alterations

**Figure 5:**
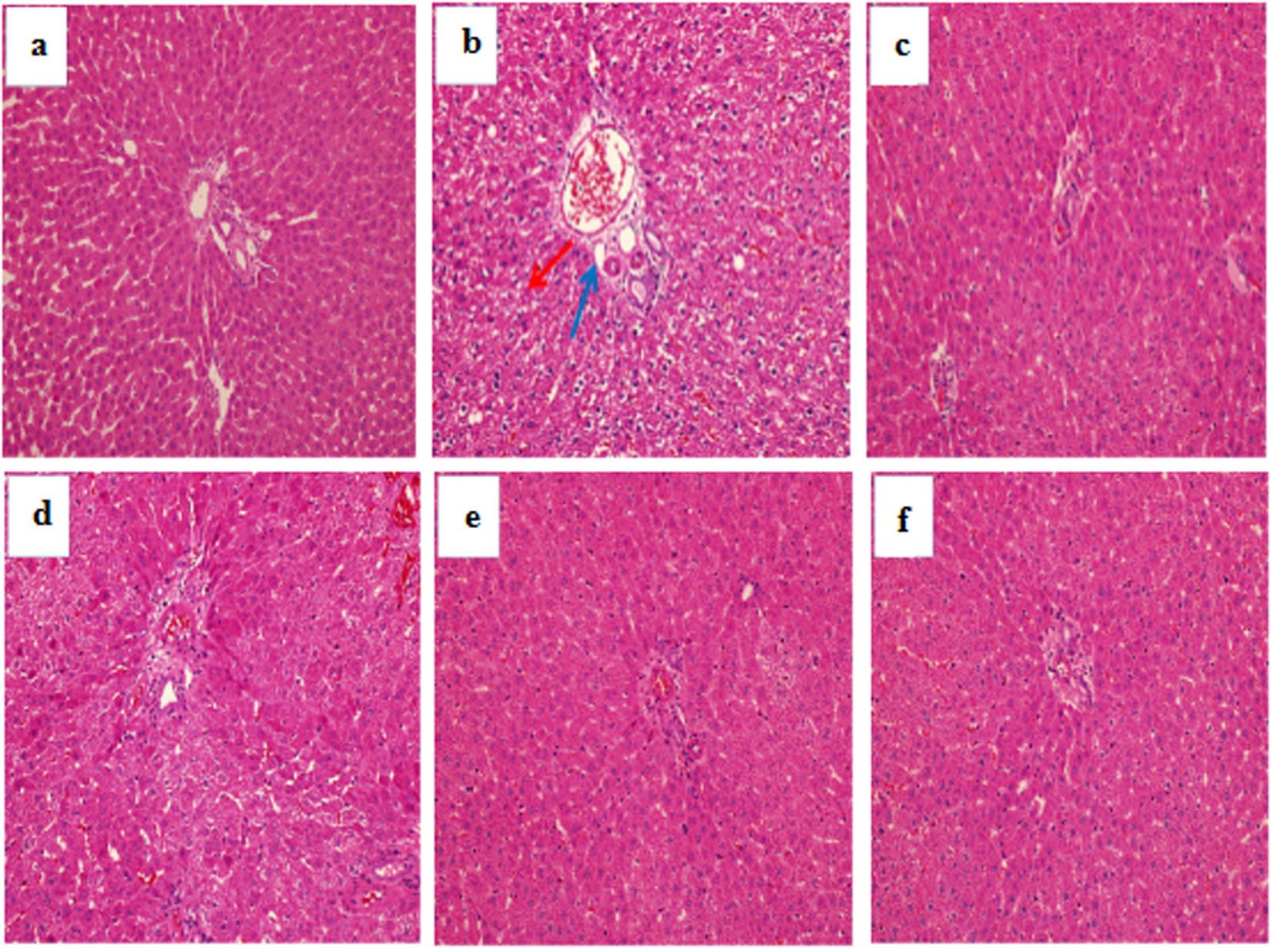
Effect of EA treatment on liver histopathological alterations (H&E staining, magnification 200×). (**a**) Blank control group; (**b**) D-gal model group; (**c**) D-gal + VE150 mg/kg; (**d**) D-gal + EA50 mg/kg; (**e**) D-gal + EA100 mg/kg; (**f**) D-gal + EA150 mg/kg. Red arrow indicated apoptosis and necrosis; Blue arrow indicated inflammatory infiltration.

As seen in Fig. 5, the histological analysis shows that the hepatic lobule of rats from the blank control group (Fig. 5a) were normal-sized and without any noticeable abnormalities in morphological structure when compared to the positive control group and the D-gal-induced ageing group (Fig. 5c). In contrast, D-gal injection resulted in moderate levels of hepatocyte apoptosis, necrosis and inflammatory cell infiltration, as observed visually (Fig. 5b). However, EA treatment noticeably ameliorated the hepatic pathological alterations, and the high-dose EA group (150 mg/kg) showed a maximum protective effect with minor changes in morphological structure and almost identical appearance to the VE and blank control groups.

**Figure 6:**
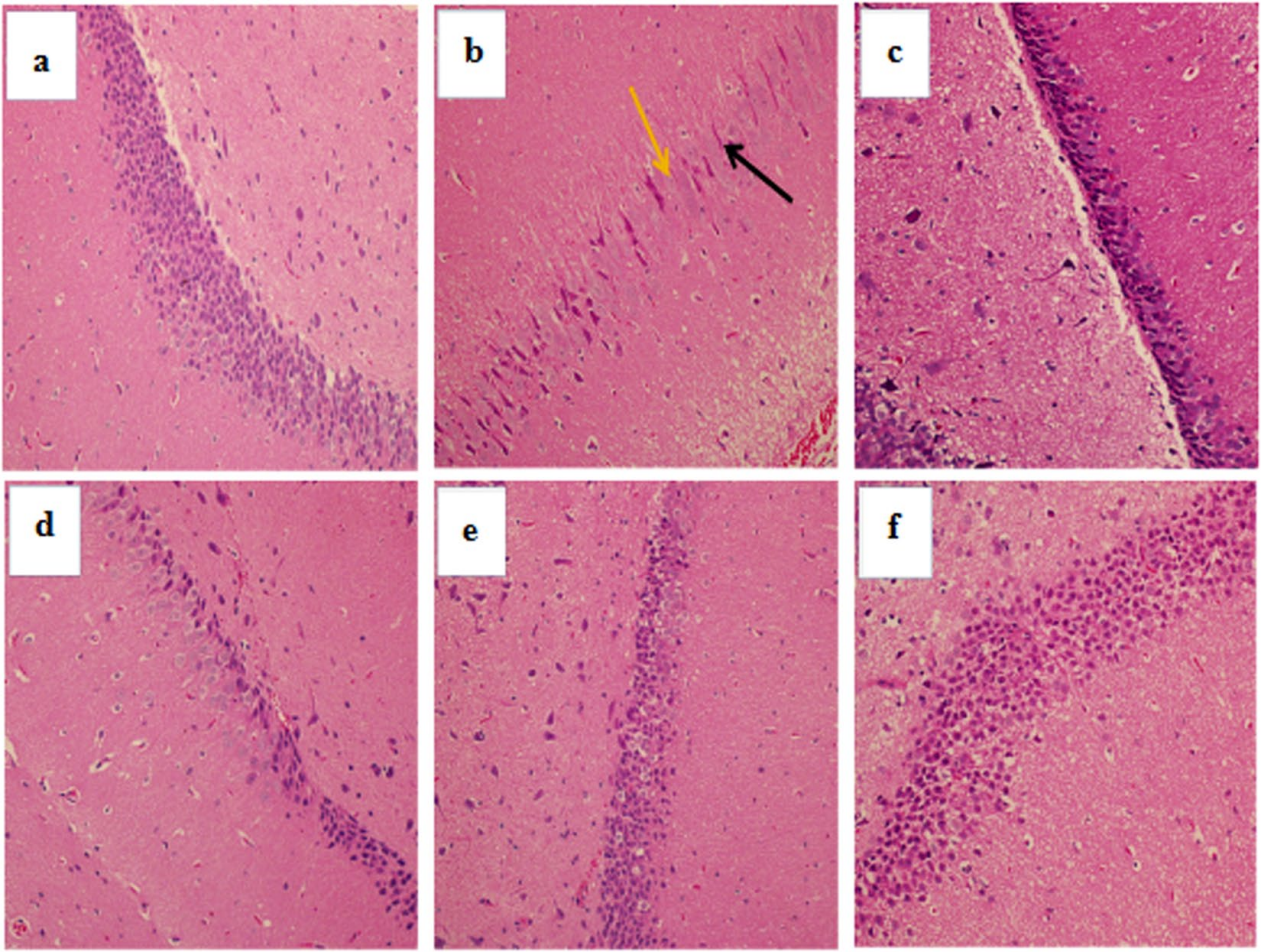
Effect of EA treatment on CA1 region of the hippocampus (H&E staining, magnification 200×). (**a**) Blank control group; (**b**) D-gal model group; (**c**) D-gal + VE150 mg/kg; (**d**) D-gal + EA50 mg/kg; (**e**) D-gal + EA100 mg/kg; (**f**) D-gal + EA150 mg/kg. Orange arrow indicated apoptosis and necrosis; Black arrow indicated irregular neurons.

As shown in Fig. 6a, no evident pathological changes were found in the brain of the blank control group under light microscopic examination. In the D-gal model group, typical characteristics of brain tissue injury, including disorganized nerve fibres with irregular neurons, and apparent fluorescent debris were found (Fig. 6b). However, there was an obvious increase in round-shaped neurons and well-organized fibres upon the intervention of EA (Fig. 6d–f). Above all, with EA treatment (150 mg/kg), the morphological structure appeared very similar to those of the blank control and positive control groups (Fig. 6c), suggesting that the extent of pathological changes in brain tissues could be improved to varying degrees by EA treatment.

### Western Blotting Analysis

**Figure 7:**
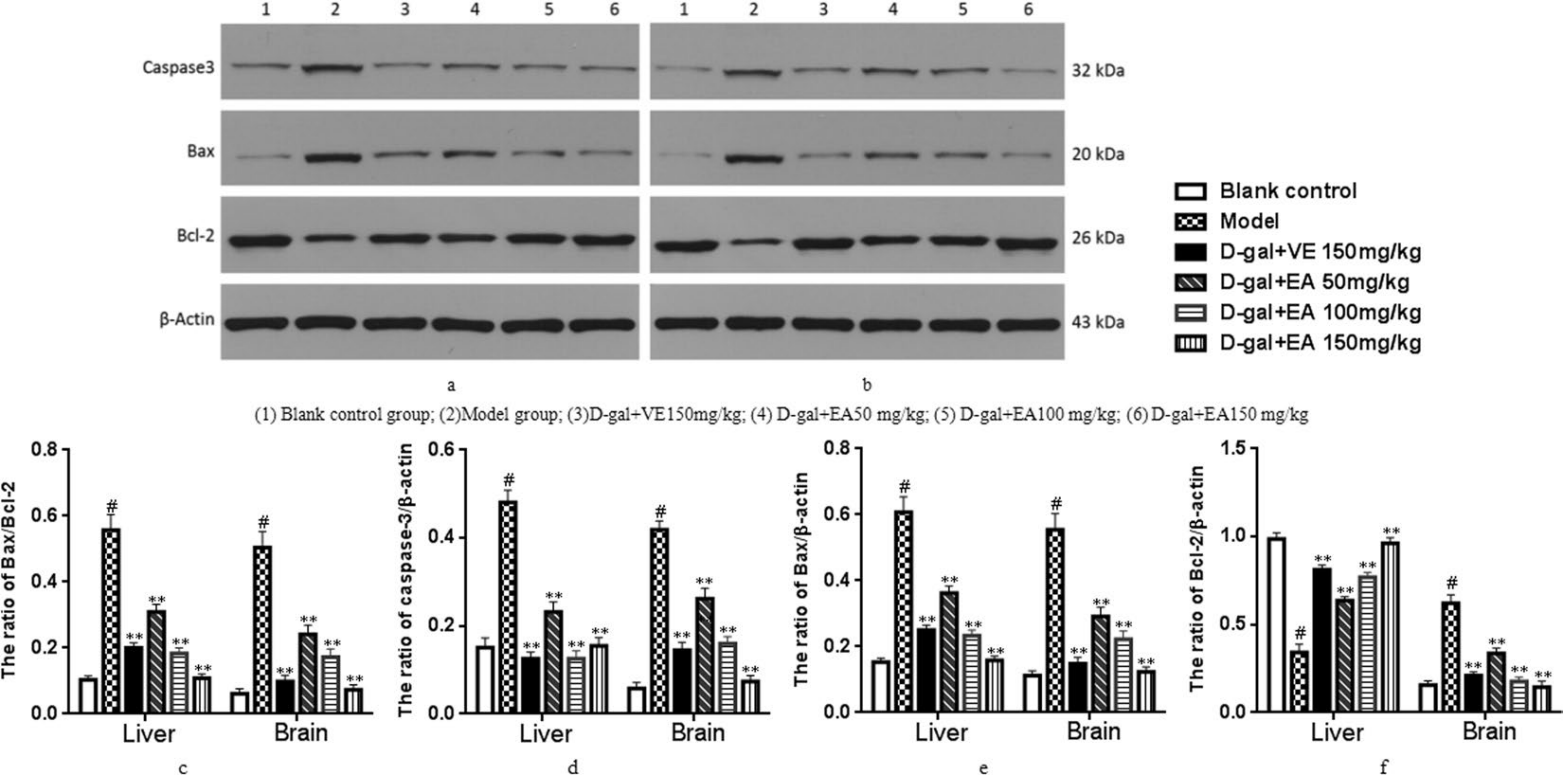
Effect of EA on the expression of Bcl-2, Bax and caspase-3 in the liver and brain. Representative western blotting of Bcl-2, Bax and caspase-3expression in liver (**a**) and brain (**b**). Quantification of Bax/Bcl-2 ration (**c**), caspase-3/β-acting ratio (**d**), Bax/β-acting ration (**e**), and Bcl-2/β-acting ration (**f**) in liver and brain. Date are presented as mean ± SD from each group(n = 8). ^#^P < 0.01 vs blank control group. **P < 0.01 and *P < 0.05 vs D-gal model group.

To determine whether the D-gal-induced ageing process in rats is related to apoptosis, we analysed the protein levels of Bcl-2, Bax and caspase-3 by Western blotting. As shown in Fig. 7, the expression of caspase-3 and the ratio of Bax/Bcl-2 significantly increased in rats after treatment with D-gal when compared to the blank control group. However, the down-regulation of these apoptosis-related proteins of the liver and brain tissues was attenuated by EA treatment in a concentration-dependent manner.

## Discussion

We confirmed that D-gal can be used to induce oxidative stress *in vivo* to mimic the ageing process of rats. Although D-gal can be changed into glucose at normal concentrations^29,30^, at high levels, D-gal is oxidized into aldehydes and hydrogen peroxide, resulting in the generation of ROS^31,32^. At the same time, D-gal can be converted into advanced substance associated with glycosylation (ASAG), and there is abundant evidence to suggest that some of the ROS in the body also come from ASAG. Accumulated evidence shows that ROS-induced oxidative stress is the key factor in inducing natural age-related changes^33^. D-gal induces ageing alterations that are similar to the normal ageing processes^34,35^. It has been confirmed in previous studies that oxidative stress could result in apoptosis, which is the main cause of many types of organ damage. Previous studies have shown that mitochondrial ROS induce the activation of a large number of mitochondrial apoptotic proteins, leading to cellular apoptosis and organ damage. The functions of two particularly important organs, the liver and the brain, decline gradually due to the attack of ROS during the ageing process^36^. First, the liver has a vitally important function of detoxification, and D-gal is mainly metabolized in the liver. Additionally, the high respiratory rate in this organ tends to expose it to ROS in daily life. Second, the brain contains the most complex tissue among all organs of the body and requires a large amount of oxygen and energy to maintain its normal activities; however, only a small amount of antioxidative enzymes are present in it^37,38^. Hence, it is widely accepted that the administration of D-gal to rats for 8 weeks can be used to establish an ageing model for anti-ageing research. The use of this model to study the effects of supplements and antioxidants has the potential to lead to new therapies for liver and brain damage due to ageing.

EA is a major ingredient of pomegranate tannins from Pericarpium Granati, and a number of studies have demonstrated that it has good antioxidant activity^39^. In our study, we aimed to demonstrate that EA can protect against D-gal-induced organ inju-ries *in vivo* during the establishment of an ageing model via subcutaneous injections of D-gal in rats for eight weeks^40^. Previous studies have demonstrated that the ageing process is characterized by changes in appearance, including significant decreases in body weight and organ indices, which can be accelerated by the administration of D-gal^41^. In present studies, it has been clearly demonstrated that the administration of Dgal caused the atrophy of the spleen and thymus, as well as the kidney, but that their corresponding organ indices could be increased after the administration of EA. Our data showed that EA has a pronounced anti-ageing effect in our D-gal-induced ageing model, and its underlying molecular mechanisms were further explored^42^.

It has been demonstrated that oxidative stress is one of the key factors responsibl e for liver damage and brain ageing via oxidative injury, as revealed by the over-production of the lipid peroxidation marker MDA and the increasing antioxidant activities of CAT, GSH-Px, SOD, and T-AOC. SOD is the first gatekeeper in the antioxidant defence system by catalysing the dismutation of the superoxide anion to oxygen and hydrogen peroxide (H_2_O_2_), the latter of which is further metabolized by CAT or GSH-Px^43–45^. T-AOC is representative of all antioxidants, and MDA is well-recognized not only as an indicator of ageing but also as the most important product of membrane lipid peroxidation, and thus, it indirectly reflects the level of cell oxidative damage. In the present work, subcutaneous injection of D-gal caused a significant decrease in antioxidant enzyme activities of CAT, GSH-Px, SOD, and T-AOC, as well as an increase in the MDA level. Interestingly, the activities of antioxidant enzymes were effectively increased by EA supplementation, which also significantly reduced the production of MDA both in the livers and brains, suggesting that EA has the potential as a pharmaceutical candidate for the prevention of ageing-related diseases^46^. The findings of our study support the view that ROS attack is closely associated with ageing and that EA could restore the antioxidant defence system by scavenging free radicals, leading to the abatement of oxidative damage in the livers and brains of D-gal-induced-ageing rats^47^.

Previous studies have demonstrated that D-gal injection can cause liver injury and dysfunction and results in gross morphological changes, as well as increased levels or activities of some serum enzymes^48^. The results of this study showed conspicuously elevated ALT and AST levels in the sera of rats after chronic injection of D-gal. Moreover, we detected hepatocyte apoptosis and inflammatory cell infiltration in hepatic tissue, as well as apoptotic cells and disorganized nerve fibres with irregular neurons in the hippocampal CA1 region^49^. These histopathological changes of the liver and brain were ameliorated after EA treatment, which suggests that EA has a protective effect on the livers and brains of rats *in vivo* during D-gal-induced ageing^50^.

There is agreement that inflammatory cytokines are involved in oxidative stress. Among the cytokines, TNF-α, IL-6 and IL-1β are essential in the development and progression of oxidative stress because these cytokines are associated with ROS and the promotion of activated NF-κB to translocate to the nucleus and regulate the expression of pro-inflammatory genes such as iNOS and COX2, which are involved in inflammatory and immune responses, and the predisposition to fibrosis, apoptosis, and acute phase responses that cause tissue and organ damage^52^. Since the levels of these inflammatory cytokines were significantly lower in model rats receiving EA compared to the D-gal-injected control group, this suggests that EA could also have a potent anti-inflammatory activity in D-gal-induced rats^53^.

Many apoptotic proteins are closely related to anti-apoptotic proteins in the ageing induced by injection of D-gal. The Bcl-2 protein is a key factor in the inhibition of apoptosis; it is a known factor in cell ageing, and its overexpression can effectively prevent the apoptosis induced by hydrogen peroxide, free radicals and microbial contamination^54^. On the other hand, the main function of Bax is to accelerate apoptosis and, together with bcl-2, regulate cell apoptosis. However, the core molecule in apoptosis is caspase-3 (cysteine protease), which is known to be a key factor of apoptosis in mammals^55^. It is commonly believed that bcl-2 acts downstream of caspase-3 activation, and thus, apoptosis is inhibited by inhibiting the activation of caspase-3, which plays an irreplaceable role in apoptosis^56^. As shown in the results, EA intervention significantly down-regulated the expression of Bcl-2 and Bax proteins while up-regulating the expression of caspase-3. The data suggest that EA treatment of model rats had anti-ap optotic effects on the D-gal-induced ageing damage on liver and brain cells by lowering the percentage of Bax and Bcl-2, which is viewed as a key factor of cell survival, through the inhibition of the activation of caspase-3^57^.

## Conclusion

In conclusion, our study evaluated the D-gal model of accelerated ageing and investigated whether EA could protect against liver and brain damage in D-gal-treated rats. Our findings support the use of the D-gal rat model to carry out ageing-related studies and revealed that EA could alleviate liver and brain damage due to ageing^58^. The mechanisms likely involved the normalization of the activities of antioxidative enzymes, the amelioration of histopathological changes, the inhibition of chronic inflammatory responses, and the attenuation of oxidative stress, as well as the modulation of the expression of ageing-related proteins^59^. These data suggest that EA has anti-ageing effects. The molecular mechanisms involved should be further explored to substantiate its anti-ageing action in future work^60^.
