# Author Correction: Antioxidative, anti-inflammatory and anti-apoptotic effects of ellagic acid in liver and brain of rats treated by D-galactose

**DOI:** 10.1038/s41598-019-55879-0

**Published:** 2019-12-10

**Authors:** Peng Chen, Fuchao Chen, Benhong Zhou

**Affiliations:** 1 Department of Pharmacy, Renmin Hospital of Wuhan University, Wuhan, 430060 Hubei Province P.R. China; 2 School of Pharmaceutical Sciences, Wuhan University, Hubei Wuhan, 430071 P.R. China; 3 Department of Pharmacy, Dongfeng Hospital, Hubei University of Medicine, Shiyan, Hubei 442008 P.R. China

Correction to: *Scientific Report* 10.1038/s41598-018-19732-0, published online 23 January 2018

This Article contains errors in Table 1 where the values listed under ‘Body weight (g)’ are incorrect.

The correct Table 1 appears below.

**Table 1:** Effects of EA on body weight and organ index in D-gal-treated rats.

Group	Body weight(g)	Organ index (mg/g)		
		Spleen index	Thymus index	Kidney index
Blank control	276.82 ± 1.55	1.87 ± 0.23	1.45 ± 0.21	10.68 ± 0.56
D-gal	212.06 ± 2.24^Δ^	1.54 ± 0.24^Δ^	1.16 ± 0.15^Δ^	8.01 ± 1.32^Δ^
VE (150 mg/kg)	274.96 ± 2.67**	1.78 ± 0.15**	1.39 ± 0.18**	9.95 ± 1.29*
EA-L (50 mg/kg)	232.4 ± 2.80*^Δ^	1.55 ± 0.28*^Δ^	1.18 ± 0.14^Δ^	8.29 ± 0.89^Δ^
EA-M (100 mg/kg)	255.03 ± 2.26**	1.86 ± 0.19**	1.45 ± 0.19**	9.10 ± 0.94*
EA-H (150 mg/kg)	282.74 ± 1.49**	1.98 ± 0.33**	1.47 ± 0.15**	9.86 ± 0.80*

Notes: Dates are given as mean ± SD (n = 8).^Δ^P < 0.05 and ^ΔΔ^P < 0.01 vs blank control

group. *P < 0.05 and **P < 0.01 vs D-gal model group.

EA-H: high-dose EA-treated group, EA-M: the medium-dose EA group

EA-L: the low-dose EA-treated group

